# Significant Correlation between Retinal Venous Tortuosity and Aqueous Vascular Endothelial Growth Factor Concentration in Eyes with Central Retinal Vein Occlusion

**DOI:** 10.1371/journal.pone.0134267

**Published:** 2015-07-27

**Authors:** Shunsuke Yasuda, Shu Kachi, Mineo Kondo, Shinji Ueno, Hiroki Kaneko, Hiroko Terasaki

**Affiliations:** 1 Department of Ophthalmology, Nagoya University Graduate School of Medicine, 65 Tsuruma-cho, Showa-ku, Nagoya 466–8550, Japan; 2 Department of Ophthalmology Mie University Graduate School of Medicine, 2–175 Edobashi, Tsu, 514–8507, Japan; Charité University Medicine Berlin, GERMANY

## Abstract

**Purpose:**

To determine whether the degree of venous tortuosity is significantly correlated with the aqueous vascular endothelial growth factor (VEGF) concentration in eyes with a central retinal vein occlusion (CRVO).

**Methods:**

We reviewed the medical records of 32 eyes of 32 patients who had macular edema due to a CRVO. All of the patients were examined at the Nagoya University Hospital and were scheduled to receive an intravitreal injection of bevacizumab (IVB) or ranibizumab (IVR) within 12 weeks of the onset of the CRVO to treat the macular edema. Aqueous humor was collected just before the IVB or IVR, and the VEGF concentration was determined by enzyme-linked immunosorbent assay (ELISA). The venous tortuosity index was calculated by dividing the length of the retinal veins by the chord length of the same segment. The correlation between the mean tortuosity index of the inferotemporal and supratemporal branches of the retinal vein and the aqueous VEGF concentration was determined.

**Results:**

The mean aqueous VEGF concentration was 384 ± 312 pg/ml with a range of 90 to 1077 pg/ml. The degree of venous tortuosity was significantly correlated with the VEGF concentration in the aqueous. (r = 0.49, *P* = 0.004), with the foveal thickness (r = 0.40, *P* = 0.02), and with the best-corrected visual acuity (r = 0.38, *P* = 0.03).

**Conclusions:**

The significant correlation between the aqueous VEGF concentration and the venous tortuosity indicates that the degree of retinal venous tortuosity can be used to identify eyes that are at a high risk of developing neovascularization.

## Introduction

A central retinal vein occlusion (CRVO) is one of the most common vision-threatening retinal vascular disorders. The circulatory blockage in a trunk of the central retinal vein leads to characteristic fundus findings such as flame-shaped intraretinal hemorrhage, venous dilation and tortuosity, and cotton wool spots.

The major causes of the decrease in vision in eyes with CRVO are macula edema and neovascular glaucoma (NVG).[1–4] It is known that increased levels of ocular vascular endothelial growth factor (VEGF) are also associated with both macular edema and NVG in CRVO eyes.[5–8] Thus, it is important to estimate the ocular VEGF concentration to determine the prognosis in eyes with a CRVO.

The Central Vein Occlusion Study (CVOS) Group reported that factors significantly associated with an increased risk of iris neovascularization (NVI) and angle neovascularization (ANV) were the visual acuity, size of the non-perfused areas detected by fluorescein angiography (FA), and the venous tortuosity.[3] At present, the size of the non-perfused area detected by FA is the most common parameter used to determine the risk of developing neovascularization. However, anaphylactic shock due to intravenous fluorescein can occur during FA although the incidence is low.[9] Also, FA is difficult to perform on patients in poor general health.

On the other hand, the venous tortuosity can be evaluated less invasively by fundus photography and optical coherence tomography (OCT).While the CVOS Group evaluated the vascular tortuosity qualitatively by the analyses of the fundus photographs, there have been reports on quantitative evaluations of the vascular tortuosity in eyes with CRVO and other retinal vascular disorders.[10–19]

Although it is known that the ocular VEGF concentration is elevated and venous tortuosity is present in eyes with CRVO, it has not been determined whether there is a significant correlation between these two factors. Thus, the purpose of this study was to determine whether the degree of venous tortuosity was significantly correlated with the aqueous VEGF concentration.

## Patients and Methods

The procedures used in this study conformed to the tenets of the World Medical Association’s Declaration of Helsinki. The collection of aqueous humor and the VEGF measurements were performed after obtaining approval of Nagoya University Hospital Ethics Review Board and a written informed consent from each patient.

### Patients

We reviewed the medical records of 32 eyes of 32 patients who had macular edema due to a CRVO. All of the patients were examined at the Nagoya University Hospital and were scheduled to receive an intravitreal injection of bevacizumab (IVB) or ranibizumab (IVR) to treat the macular edema within 12 weeks of the onset of the CRVO. Aqueous humor was collected just before the IVB or IVR. From December of 2008 to June of 2010, 15 patients received IVB and from August 2013 to September 2014, 17 patients received IVR. Patients with diabetic retinopathy were excluded. Fellow eyes with a medical history of retinal disease were excluded from the analyses. Thirteen of 15 patients who received IVB were included in our earlier publication.[20]

### Calculation of venous tortuosity index

**Fig 1:**
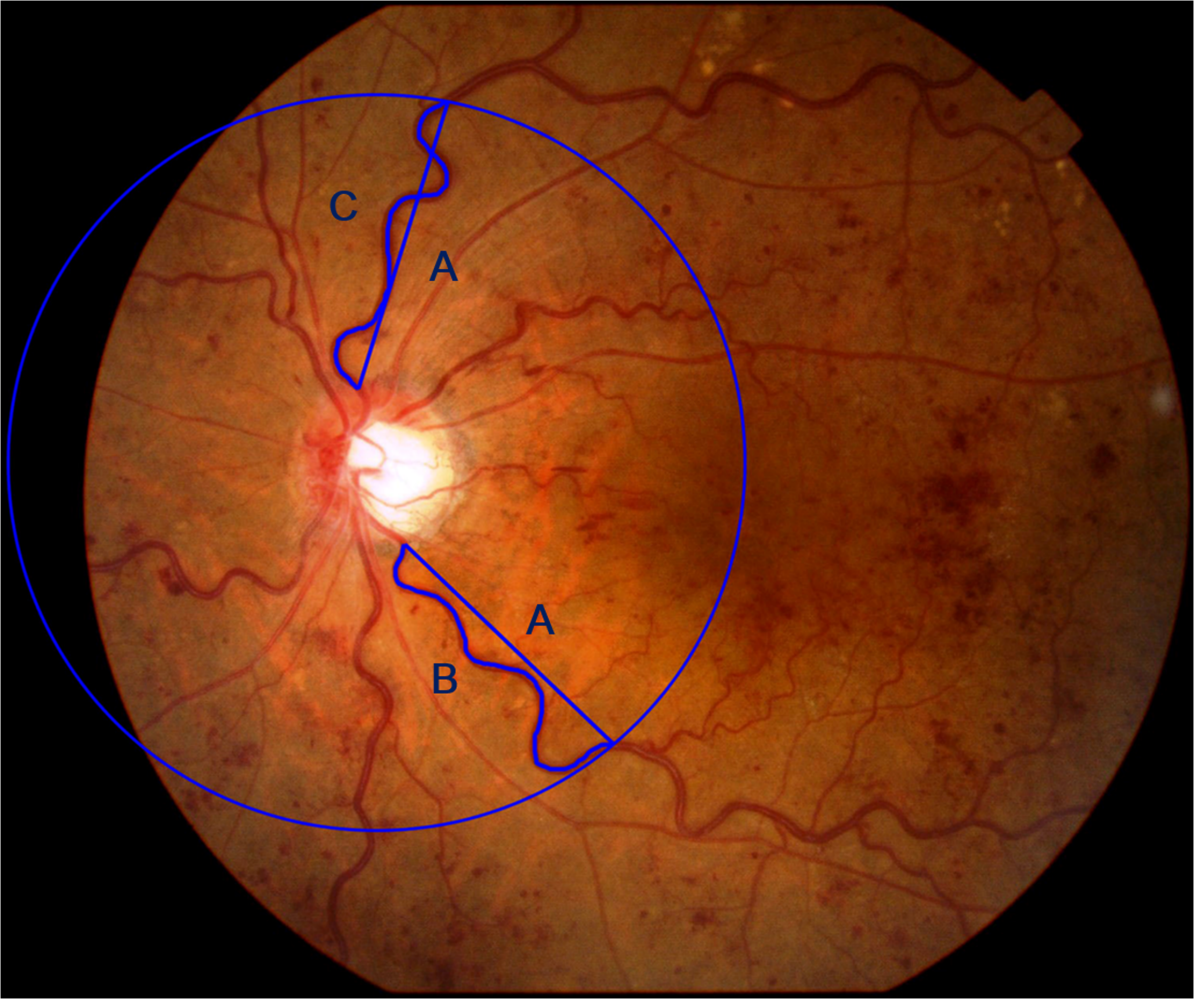
Calculation of venous tortuosity index. Measurements of superior and inferior venous arcades were obtained starting from the optic disc margin to the crossing point of a circle (A) whose diameter is the distance from the center of optic disc to the fovea. The course of the veins were traced using Photoshop (Adobe Systems, Inc. Ca, USA). NIH ImageJ software was used to measure the lengths of the vein (A) and chord (B and C) of the vessels. The venous tortuosity index was calculated by dividing the length of the retinal veins by the chord length of the same segment (B/ A and C/A). The average of the venous tortuosity ((B/A + C/A)/2) was calculated to obtain the venous tortuosity index.

Fundus photographs were obtained with a Topcon fundus camera (TRC-NW7SF, Topcon, Tokyo, Japan) or with a Canon fundus camera (CF-60DSi, Canon, Tokyo, Japan). The course of the veins was traced using the Photoshop software (Adobe Systems, Inc. Ca, USA). Measurements of the superior and inferior venous arcades were obtained starting from the optic disc margin to the crossing point of a circle (A) whose diameter was the distance from the center of optic disc to the fovea. NIH ImageJ software was used to measure the length of the tortuous vein (A) and length of the chord (B and C) of the vessels. The venous tortuosity was calculated by dividing the arc length of the retinal veins by the chord length of the same segment (B/ A and C/A) as reported (Fig 1).[17] The average of the venous tortuosity of all of the veins was calculated to obtain the venous tortuosity index.

### Intravitreal injection of bevacizumab or ranibizumab and collection of aqueous humor

The eyes were anesthetized with topical 1% tetracaine, and the fornices were irrigated with 10% providone-iodine. A mean volume of 0.1 ml of aqueous humor was collected by anterior chamber paracentesis with a 27-gauge needle attached to a 1 ml syringe. Each patient then received an intravitreal injection of 1.25 mg/0.05 ml of bevacizumab or ranibizumab using a 30-gauge needle inserted through the sclera 3.5 mm from the limbus. Moxifloxacin (0.5% ophthalmic solution, VEGAMOX; Alcon Japan Ltd., Tokyo, Japan) was applied topically for three days after the injection.

### Measurement of VEGF level in aqueous by ELISA

The aqueous samples were stored at -80° C until use. The concentration of VEGF was measured by enzyme-linked immunesorbent assay (ELISA) with a commercial kit (Quantikine:R&D Systems Inc., Minneapolis, MN), which measures the concentrations of both human VEGF121 and VEGF165.[20,21]

### Best-corrected visual acuity (BCVA)

The best-corrected visual acuity (BCVA) was measured at 5 m with a Landolt chart. The decimal values were converted to the logarithm of the minimum angle of resolution (logMAR) units for the statistical analyses.

### Foveal Thickness

The foveal thickness was determined by optical coherence tomography (OCT; Cirrus model; Carl Zeiss Meditec, Dublin, CA). After the patients’ pupils were fully dilated with 0.5% tropicamide and 0.5% phenylephrine (Mydrin-P, Santen Co., Japan), 6 mm vertical and horizontal scans were made through the fovea. The average of the foveal thickness for the vertical and horizontal scans was used as the foveal thickness. A manual method was used to place the cursors on the OCT images to measure the foveal thickness because it has been reported that the automatic measurements of the foveal thickness often failed to identify the outer border of the neural retina accurately.[22,23]

### Statistical analyses

The Pearson correlation coefficient was used to determine the correlations between the different parameters. Paired *t* tests were used to determine whether the venous tortuosity index of the affected eyes were significantly different from that of the fellow eyes. A *P* value <0.05 was considered significant. The SPSS version17.0J (for Windows, SPSS Inc., Chicago, IL) was used for all statistical analyses.

## Results

### Demographics and ocular VEGF concentrations of patients

Thirty-two eyes of 32 patients were studied. There were 21 men and 11 women, and their mean age was 66.2 ± 13.3 years with a range of 45 to 85 years. The mean duration of the symptoms before the collection of aqueous humor was 7.0 weeks (2 to 12 weeks). None of the eyes had neovascularization of the angle or on the iris. At the time of the IVB or IVR, the mean visual acuity was 0.76 logMAR units with a range of 0.10 to 2.00 logMAR units. The mean foveal thickness determined by optical coherence tomography (OCT) was 686 ± 253 μm with a range of 330 to 1200 μm (Table 1). The mean VEGF concentration in the aqueous humor was 384 pg/ml with a range of 90 to 1077 pg/ml prior to the IVB or IVR.

**Table 1:** Characteristics of patients with CRVO.

Number of eyes	32
Age (year)*	66.2 ± 13.3 (26–85)
Sex (men/women)	21/ 11
Duration of symptoms before IVB/IVR (weeks)*	7.3 ± 3.5 (2–12)
pre-IVB/IVR photocoagulation (eyes)	0
pre-IVB/IVR visual acuity (logMAR)*	0.75 ± 0.50 (0.10–2.00)
pre-IVB/IVR foveal thickness (μm)*	686 ± 253 (330–1625)

*Data are expressed as mean ± SD (range)

### Comparisons of venous tortuosity index between affected and fellow eyes

We compared the venous tortuosity index of the affected eyes to that of the fellow eyes using paired *t* tests. The mean venous tortuosity index of the affected eyes was significantly larger than that of the fellow eyes (1.173 ± 0.056 vs 1.095 ± 0.039; *P* <0.0001).

### Correlation between venous tortuosity and concentration of VEGF in aqueous humor

**Fig 2:**
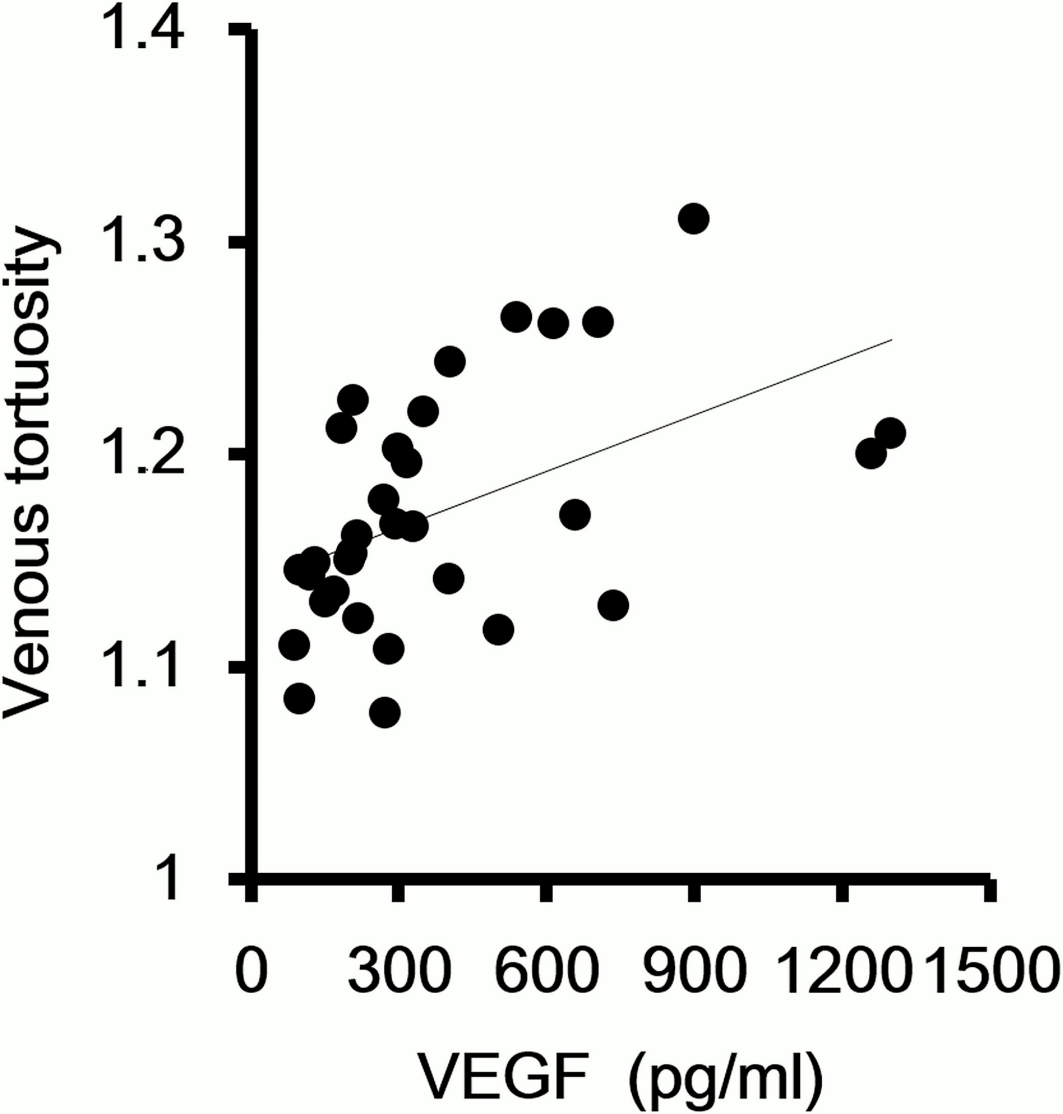
Correlation between venous tortuosity and concentration of VEGF in the aqueous humor. The degree of venous tortuosity is significantly correlated with the VEGF concentration in the aqueous. (r = 0.49, *P* = 0.004)

The coefficient of correlation between the venous tortuosity index and the VEGF concentration in the aqueous humor was determined by the Pearson correlation coefficient. The venous tortuosity index was significantly correlated with the VEGF concentration in the aqueous. (r = 0.49, *P* = 0.004, Fig 2).

### Correlation between venous tortuosity index and foveal thickness and visual acuity

**Fig 3:**
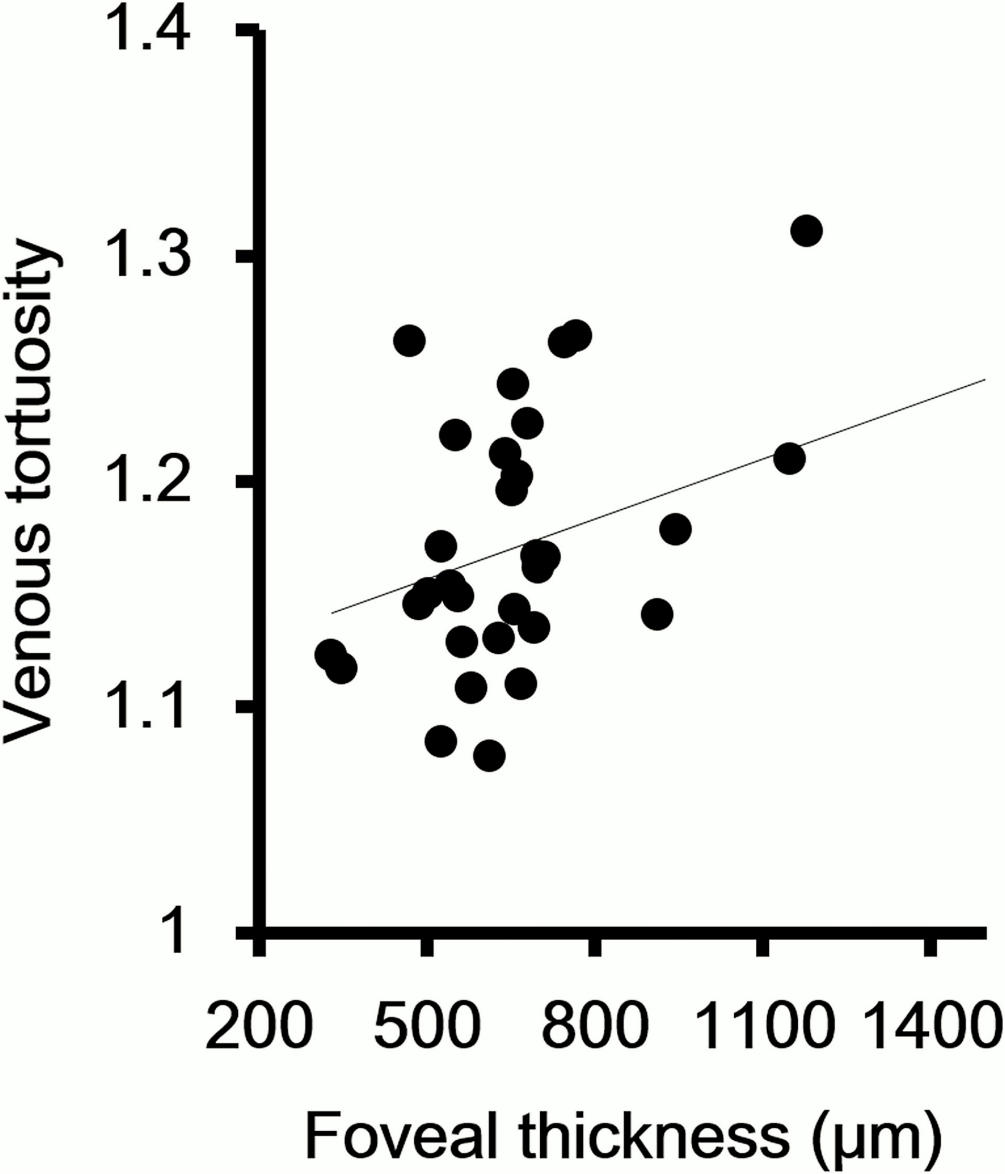
Correlation between venous tortuosity index and foveal thickness. The degree of venous tortuosity is significantly correlated with the foveal thickness. (r = 0.40, *P* = 0.02)

**Fig 4:**
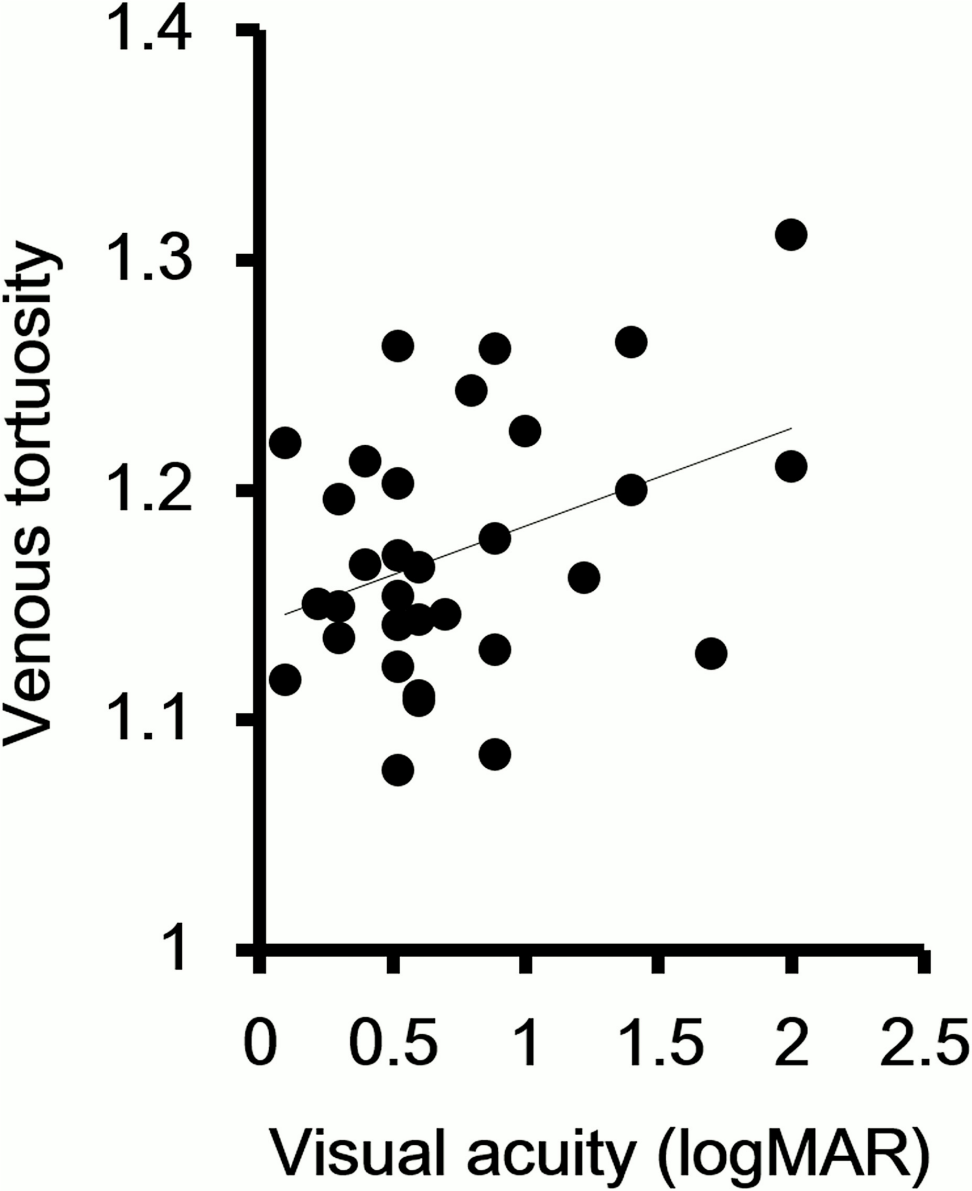
Correlation between venous tortuosity index and visual acuity. The degree of venous tortuosity is significantly correlated with the visual acuity. (r = 0.38, *P* = 0.03).

The venous tortuosity index was significantly correlated with the foveal thickness. (r = 0.40, *P* = 0.02, Fig 3). Because it is known that a low visual acuity is a predictive factor for the development of NVI, we examined whether there was a significant correlation between the visual acuity and the venous tortuosity index. Our analyses showed that the degree of venous tortuosity was significantly correlated with the visual acuity (r = 0.38, *P* = 0.03, Fig 4).

## Discussion

Our results showed that the concentration of VEGF in the aqueous of eyes with a CRVO was significantly correlated with the degree of venous tortuosity. There have been earlier studies that evaluated the degree of vascular tortuosity in different retinal vascular disorders quantitatively especially in eyes with retinopathy of prematurity (ROP) for the diagnosis of plus diseases.[10,11,15] In CRVO eyes, Muraoka et al. measured the anterio-posterior venous tortuosity in the OCT images. They reported that the degree of anterior-posterior tortuosity was significantly correlated with the retinal perfusion status.[13] Ferrara et al. evaluated the venous tortuosity using the same method as we did in this study, and they reported that the venous tortuosity was significantly decreased after IVB in CRVO eyes. They reported that the reduction in venous tortuosity after VEGF blockage by IVB implied an improvement of venous flow, and that high levels of VEGF might result in the proliferation of intraluminal capillary endothelial cell and the reduction of venous flow.[17] The method we used can be performed relatively easily using fundus photographs, Photoshop, and NIH ImageJ software.

The mechanism of how CRVO leads to venous tortuosity is still unclear. However, the significant correlation between the venous tortuosity and intraocular VEGF concentration suggests that the venous tortuosity is correlated with the degree of circulatory disorder and retinal ischemia.

Our results are compatible with a previous report that assessed the venous tortuosity qualitatively and reported that venous tortuosity was a risk factors of NVI/ANV.[3] Although it is possible to evaluate the non-perfused areas using FA to avoid NVG, we suggest that it is possible to predict the ocular VEGF concentration noninvasively by evaluating the venous tortuosity. The increased level of ocular VEGF is associated with NVG in CRVO eyes.[7] Thus, it is clinically important to estimate the ocular VEGF level during the course of CRVO.

There are two limitations in our study. The first was our small sample size, and the second was that we did not know the chronological changes in the aqueous VEGF concentration during the natural course of CRVO. It is difficult from an ethical point of view to collect aqueous samples during the natural course of CRVO. Because it has been reported that neovascular glaucoma usually occurs in the first 8 months,[4] we assumed that the ocular VEGF level will reach its peak at a relatively early stage of CRVO. Thus, we have excluded cases whose onset of CRVO was >12 weeks.

In conclusion, our results showed that the VEGF concentration in the aqueous of eyes with a CRVO was significantly correlated with the venous tortuosity. Because eyes that develop iris and angle neovascularization have high concentrations of aqueous VEGF, our findings indicate that the degree of retinal venous tortuosity can be used to identify eyes that are at a high risk of developing neovascularization.

## References

[1] McIntosh RL, Rogers SL, Lim L, Cheung N, Wang JJ, Mitchell P, et al Natural history of central retinal vein occlusion: an evidence-based systematic review. Ophthalmology. 2010;117(6):1113–23 e15. Epub 2010/05/01. 10.1016/j.ophtha.2010.01.060 20430446

[2] Hayreh SS, Podhajsky PA, Zimmerman MB. Natural history of visual outcome in central retinal vein occlusion. Ophthalmology. 2011;118(1):119–33 e1–2. Epub 2010/08/21. 10.1016/j.ophtha.2010.04.019 20723991 PMC2989417

[3] Natural history and clinical management of central retinal vein occlusion. The Central Vein Occlusion Study Group. Archives of ophthalmology. 1997;115(4):486–91. Epub 1997/04/01. 9109757 10.1001/archopht.1997.01100150488006

[4] Hayreh SS, Rojas P, Podhajsky P, Montague P, Woolson RF. Ocular neovascularization with retinal vascular occlusion-III. Incidence of ocular neovascularization with retinal vein occlusion. Ophthalmology. 1983;90(5):488–506. Epub 1983/05/01. 6192376 10.1016/s0161-6420(83)34542-5

[5] Aiello LP, Avery RL, Arrigg PG, Keyt BA, Jampel HD, Shah ST, et al Vascular endothelial growth factor in ocular fluid of patients with diabetic retinopathy and other retinal disorders. The New England journal of medicine. 1994;331(22):1480–7. Epub 1994/12/01. 10.1056/nejm199412013312203 7526212

[6] Pe'er J, Folberg R, Itin A, Gnessin H, Hemo I, Keshet E. Vascular endothelial growth factor upregulation in human central retinal vein occlusion. Ophthalmology. 1998;105(3):412–6. Epub 1998/03/21. 10.1016/s0161-6420(98)93020-2 9499769

[7] Tripathi RC, Li J, Tripathi BJ, Chalam KV, Adamis AP. Increased level of vascular endothelial growth factor in aqueous humor of patients with neovascular glaucoma. Ophthalmology. 1998;105(2):232–7. Epub 1998/02/28. 9479280 10.1016/s0161-6420(98)92782-8

[8] Noma H, Funatsu H, Mimura T, Harino S, Sone T, Hori S. Increase of vascular endothelial growth factor and interleukin-6 in the aqueous humour of patients with macular oedema and central retinal vein occlusion. Acta ophthalmologica. 2010;88(6):646–51. 10.1111/j.1755-3768.2009.01524.x 19563372

[9] Ha SO, Kim DY, Sohn CH, Lim KS. Anaphylaxis caused by intravenous fluorescein: clinical characteristics and review of literature. Internal and emergency medicine. 2014;9(3):325–30. Epub 2013/12/03. 10.1007/s11739-013-1019-6 24293212

[10] Wilson CM, Cocker KD, Moseley MJ, Paterson C, Clay ST, Schulenburg WE, et al Computerized analysis of retinal vessel width and tortuosity in premature infants. Investigative ophthalmology & visual science. 2008;49(8):3577–85. Epub 2008/04/15. 10.1167/iovs.07-1353 18408177

[11] Thyparampil PJ, Park Y, Martinez-Perez ME, Lee TC, Weissgold DJ, Berrocal AM, et al Plus disease in retinopathy of prematurity: quantitative analysis of vascular change. American journal of ophthalmology. 2010;150(4):468–75 e2. Epub 2010/07/21. 10.1016/j.ajo.2010.04.027 20643397 PMC2945436

[12] Sasongko MB, Wong TY, Nguyen TT, Cheung CY, Shaw JE, Wang JJ. Retinal vascular tortuosity in persons with diabetes and diabetic retinopathy. Diabetologia. 2011;54(9):2409–16. Epub 2011/06/01. 10.1007/s00125-011-2200-y 21625945

[13] Muraoka Y, Tsujikawa A, Kumagai K, Akagi-Kurashige Y, Ogino K, Murakami T, et al Retinal vessel tortuosity associated with central retinal vein occlusion: an optical coherence tomography study. Investigative ophthalmology & visual science. 2014;55(1):134–41. 10.1167/iovs.13-13201 24327610

[14] Mohsenin A, Mohsenin V, Adelman RA. Retinal vascular tortuosity in obstructive sleep apnea. Clinical ophthalmology. 2013;7:787–92. 10.2147/OPTH.S41795 23641149 PMC3639720

[15] Keck KM, Kalpathy-Cramer J, Ataer-Cansizoglu E, You S, Erdogmus D, Chiang MF. Plus disease diagnosis in retinopathy of prematurity: vascular tortuosity as a function of distance from optic disk. Retina (Philadelphia, Pa). 2013;33(8):1700–7. Epub 2013/03/30. 10.1097/IAE.0b013e3182845c39 PMC3759352 23538582

[16] Hart WE, Goldbaum M, Cote B, Kube P, Nelson MR. Measurement and classification of retinal vascular tortuosity. International journal of medical informatics. 1999;53(2–3):239–52. Epub 1999/04/08. 10193892 10.1016/s1386-5056(98)00163-4

[17] Ferrara DC, Koizumi H, Spaide RF. Early bevacizumab treatment of central retinal vein occlusion. American journal of ophthalmology. 2007;144(6):864–71. 10.1016/j.ajo.2007.07.038 17916320

[18] Cheung CY, Zheng Y, Hsu W, Lee ML, Lau QP, Mitchell P, et al Retinal vascular tortuosity, blood pressure, and cardiovascular risk factors. Ophthalmology. 2011;118(5):812–8. Epub 2010/12/15. 10.1016/j.ophtha.2010.08.045 21146228

[19] Bhuiyan A, Nath B, Ramamohanarao K, Kawasaki R, Wong TY. Automated analysis of retinal vascular tortuosity on color retinal images. Journal of medical systems. 2012;36(2):689–97. Epub 2010/08/13. 10.1007/s10916-010-9536-6 20703661

[20] Yasuda S, Kachi S, Kondo M, Ushida H, Uetani R, Terui T, et al Significant correlation between electroretinogram parameters and ocular vascular endothelial growth factor concentration in central retinal vein occlusion eyes. Investigative ophthalmology & visual science. 2011;52(8):5737–42. 10.1167/iovs.10-6923 21642626

[21] Nonobe NI, Kachi S, Kondo M, Takai Y, Takemoto K, Nakayama A, et al Concentration of vascular endothelial growth factor in aqueous humor of eyes with advanced retinopathy of prematurity before and after intravitreal injection of bevacizumab. Retina (Philadelphia, Pa). 2009;29(5):579–85. Epub 2009/05/12. 10.1097/IAE.0b013e3181a3b848 19430279

[22] Costa RA, Calucci D, Skaf M, Cardillo JA, Castro JC, Melo LA Jr., et al Optical coherence tomography 3: Automatic delineation of the outer neural retinal boundary and its influence on retinal thickness measurements. Investigative ophthalmology & visual science. 2004;45(7):2399–406. Epub 2004/06/30. 15223823 10.1167/iovs.04-0155

[23] Kakinoki M, Sawada O, Sawada T, Kawamura H, Ohji M. Comparison of macular thickness between Cirrus HD-OCT and Stratus OCT. Ophthalmic surgery, lasers & imaging: the official journal of the International Society for Imaging in the Eye. 2009;40(2):135–40. Epub 2009/03/27. 10.3928/15428877-20090301-09 19320302

